# Circadian Rhythm and Sleep Disruption: Causes, Metabolic Consequences, and Countermeasures

**DOI:** 10.1210/er.2016-1083

**Published:** 2016-10-20

**Authors:** Gregory D. M. Potter, Debra J. Skene, Josephine Arendt, Janet E. Cade, Peter J. Grant, Laura J. Hardie

**Affiliations:** Division of Epidemiology and Biostatistics (G.D.M.P., L.J.H.), LIGHT Laboratories, University of Leeds, Leeds LS2 9JT, United Kingdom; Chronobiology Section (D.J.S., J.A.), Faculty of Health and Medical Sciences, University of Surrey, Guildford GU2 7XH, United Kingdom; Nutritional Epidemiology Group (J.E.C.), School of Food Science and Nutrition, University of Leeds, Leeds LS2 9JT, United Kingdom; and Division of Cardiovascular & Diabetes Research (P.J.G.), LIGHT Laboratories, University of Leeds, Leeds LS2 9JT, United Kingdom

## Abstract

Circadian (∼24-hour) timing systems pervade all kingdoms of life and temporally optimize behavior and physiology in humans. Relatively recent changes to our environments, such as the introduction of artificial lighting, can disorganize the circadian system, from the level of the molecular clocks that regulate the timing of cellular activities to the level of synchronization between our daily cycles of behavior and the solar day. Sleep/wake cycles are intertwined with the circadian system, and global trends indicate that these, too, are increasingly subject to disruption. A large proportion of the world's population is at increased risk of environmentally driven circadian rhythm and sleep disruption, and a minority of individuals are also genetically predisposed to circadian misalignment and sleep disorders. The consequences of disruption to the circadian system and sleep are profound and include myriad metabolic ramifications, some of which may be compounded by adverse effects on dietary choices. If not addressed, the deleterious effects of such disruption will continue to cause widespread health problems; therefore, implementation of the numerous behavioral and pharmaceutical interventions that can help restore circadian system alignment and enhance sleep will be important.

- Introduction
- The Circadian System
  - The suprachiasmatic nuclei
  - Clock genes
  - Post-transcriptional clock regulation
  - Non-transcriptional oscillators
  - Internal synchrony
  - Temporal partitioning of physiology
- Consequences of Circadian Rhythm and Sleep Disruption
  - Sleep restriction and sleep deprivation
  - Limited daytime light exposure
  - Increased light exposure at night
  - Shift work
- Circadian System Genetics and Metabolism
- Countermeasures Against the Metabolic Consequences of Circadian Rhythm and Sleep Disruption
- Conclusions

## I. Introduction

Mankind's historic fascination with the temporal world has taken many forms, from pilgrimages to Stonehenge at the time of the summer solstice for over 5000 years to fanciful notions about time travel. This world has shaped life by means of such rhythmic environmental stimuli as the 24-hour light/dark (LD) cycle, stimuli that have made organisms evolve their own timing systems to anticipate and adapt to daily and seasonal cycles. Thomas Edison's seminal invention of the electric light bulb in 1879 brought unprecedented possibilities, and the American inventor is attributed with once remarking, “The doctor of the future will give no medicine, but will instruct his patient in the care of the human frame, in diet, and in the cause and prevention of disease.” Little was he aware that mistimed use of his great gift to the world is now one of several human-imposed environmental changes that predispose us to many diseases by way of circadian rhythm and sleep disruption (Table 1, Glossary). The purposes of this review are to detail our current knowledge about the causes and metabolic consequences of such disruption and to highlight strategies to counteract these consequences. Although studies of other animals have been pivotal in furthering our understanding of the regulation of the circadian system and sleep, it may be premature to extrapolate findings from commonly studied model organisms, particularly nocturnal ones, to our own diurnal species. Therefore, this review focuses on human studies of healthy participants where possible, beginning with observational studies that provide insights into the prevalence of circadian rhythm and sleep disruption.

**Table 1.** Glossary

Chronobiotic	An agent capable of modifying a biological rhythm's amplitude (the difference between a rhythm's acrophase [peak] or bathyphase [trough] and its mean value), period (the time after which a phase of the rhythm oscillation recurs) or phase (the instantaneous state of an oscillation within a period).
Chronotype	An individual's phase angle of entrainment (for example, the timing of core body temperature nadir relative to dawn).
Circadian rhythm	An endogenous rhythm with a period of approximately 24 hours that is entrainable, persists in the absence of external time cues (1), and is temperature compensated. For most biological systems, the Q10 temperature coefficient—a measure of the rate of change of a biological or chemical system after increasing the temperature by 10°C—is approximately 2; for the circadian system, however, it is close to 1, allowing stable circadian rhythms in different thermal environments (2). Circadian time (CT) is synonymous with internal time and is the time of one full circadian period. CT 0 is commonly subjective dawn.
Circadian rhythm disruption	Disruption of biological timing. This can occur from the level of the molecular clock (that temporally regulates cellular activities) to misalignment between behavioral and environmental cycles. Circadian rhythm disruption produces a loss of characteristic phase relationships in oscillatory subsystems, the nature of which is ill-defined.
Constant routine	Constant routine experimental protocols attempt to enforce unchanging behavioral and environmental conditions, such as constant dim lighting, evenly spaced isoenergetic snack consumption, semi-recumbent posture, and wakefulness, to distinguish true endogenous circadian rhythms from diurnal rhythms arising from both endogenous and exogenous factors.
Entrainment	Coupling of an endogenous rhythm to a zeitgeber, such that the oscillations have the same frequency (synchronization) or frequencies that are integral multiples (frequency demultiplication) (3). Entrainment is achieved when internal period (τ) matches external period (T). Because the mean free-running human τ is approximately 24.2 hours (4, 5), circadian rhythms must be entrained to the 24-hour LD cycle daily. A short or long free-running τ typically entrains earlier or later, respectively.
Forced desynchrony	Forced desynchrony experimental protocols use LD cycles outside the range of entrainment to distribute sleep and wakefulness across the circadian cycle and, hence, uncouple effects of behavioral cycles from effects of circadian rhythms. An example protocol might involve 28-hour sleep/wake cycles, three cycles of which would produce approximately 180° misalignment between the circadian clock and LD cycle.
Peripheral clocks	Peripheral clocks comprise extra-suprachiasmatic nuclei (SCN) brain clocks and clocks in peripheral tissues. These clocks produce circadian rhythms in local tissue processes.
Sleep disruption	By sleep disruption, we refer to externally mediated changes in sleep continuity, timing, or duration (restriction entails reduced sleep duration, whereas deprivation is the absence of sleep).
Zeitgeber	The oscillation force that entrains a biological rhythm (3). The 24-hour LD cycle is the primary zeitgeber for humans. High amplitude, relatively consistently timed zeitgebers help ensure stable entrainment (for example, high light exposure during the day and minimal light exposure at night). Zeitgeber time (ZT) is the duration of one zeitgeber cycle. ZT 0 is commonly dawn or the beginning of the warm phase.

Table 2 provides an overview of sources and mechanisms of circadian rhythm disruption, one source of which is shift work. Because shift workers often work during the night (the rest phase for humans, as diurnal organisms), they are at particular risk of circadian rhythm and sleep disruption (6–9). Shift workers are also predisposed to other health disorders, such as gastrointestinal issues (10), and shift work exposure is related to risk of some diseases in a dose-response fashion, including breast cancer and metabolic syndrome (11, 12). Findings from observational studies also suggest that circadian rhythm and sleep disruption are intertwined with some of these disorders: compared to day shift workers matched for body mass index (BMI), for example, some of the adverse metabolic consequences experienced by night shift workers are coincident with sleep disturbances (13). Because shift workers make up approximately 17% of the European workforce and approximately 15% of the U.S. workforce (14, 15), the societal implications of the health consequences of shift work are substantial.

**Table 2.** Sources and Primary Mechanisms of Circadian Rhythm Disruption

Source	Mechanism				
	Environmental LD Cycle Disruption	Behavioral		Biological	
		Feeding/Fasting Cycle Disruption	Rest/Activity Cycle Disruption	Genetic Disruption (eg, Clock Gene Mutations)	Physiological Disruption (eg, Retinal Dysfunction)
Work schedules (eg, shift work, social jetlag, early school start times)	✓	✓	✓	×	×
Jetlag	✓	✓	✓	×	×
Unusual photoperiods (eg, polar regions)	✓	×	×	×	×
Circadian rhythm sleep/wake disorders (eg, non-24-hour sleep/wake disorder)	✓	×	×	✓	✓
Senescence	×	×	×	×	✓
Disease states (eg, Alzheimer's, Smith-Magenis syndrome)	×	×	×	✓	✓

Like shift work, jetlag induces circadian rhythm and sleep disruption. Although the health consequences of frequent jetlag are equivocal (16), any deleterious effects of jetlag-induced circadian rhythm and sleep disruption will become more widespread because it has been estimated that there will be approximately 831 million more air-bound passengers globally in 2016 compared to 2011 (17).

Whereas shift work and jetlag entail overt disruption to the circadian system and sleep, even “normal” working hours can result in more subtle circadian rhythm misalignment and sleep restriction, particularly among evening chronotypes. This is because many individuals use alarms to produce wakefulness when sleep would otherwise occur. Hence, bedtimes tend to differ between workdays and non-workdays, and a discrepancy of at least 1 hour between midsleep time on workdays and non-workdays affects approximately 87% of Northern Europeans. This phenomenon is often termed “social jetlag” and is associated with obesity and behavioral ramifications, such as increased alcohol consumption and smoking (18, 19). Similarly, greater intraindividual sleep timing variability has been linked to higher fat mass and lower lean mass (20), as well as cardiometabolic disease risk factors like insulin resistance (21, 22).

Alarm clock use contributes to pervasive short sleep duration among adults, and analysis of approximately 250 000 self-reports of sleep worldwide suggests that sleep duration on workdays has declined by approximately 3.7 minutes per year in the last decade (18), the significance of which is that sleep duration is associated with numerous chronic diseases. For example, sleep duration has a U-shaped association with type 2 diabetes mellitus (T2DM) prevalence. The mechanisms underlying the association between short sleep and increased T2DM risk will be discussed subsequently; the reason why long sleep is associated with increased T2DM risk is more contentious, but low socioeconomic status, depression, and other comorbidities are thought to contribute to this relationship (23).

An inverse association between sleep duration and adiposity is evident in observational studies (24, 25). In addition, findings from a recent meta-analysis indicate that a negative association between sleep duration and waist circumference is apparent (26). Importantly, fat mass distribution is particularly salient to metabolic health, with central obesity linked to increased risk of several diseases. The relationship between sleep duration and adiposity is not limited to adults. Because chronotype delays during growth and is latest on reaching physical maturity, enforcing early school starts disrupts sleep timing and duration during adolescence, a critical developmental period (27) and, once more, short sleep during this period is prospectively associated with obesity development (28). Relationships between sleep and adiposity are related to eating behaviors, and links between circadian rhythm and sleep disruption, dietary habits, and fat mass are further apparent in less common disorders like night eating syndrome (29).

In contrast to effects of jetlag and working demands on the circadian system and sleep, some individuals are at increased risk of circadian rhythm and sleep disruption regardless of cultural changes. At times this is environmentally driven. All 24 time zones converge at the Poles, for example, where low amplitude LD cycles and extreme temperatures are associated with health ramifications (30). In other instances, underlying pathologies are at fault. This is true in the case of circadian rhythm disruption in blind individuals without light perception (31), most of whom experience non-24-hour sleep/wake rhythm disorder in which sleep quality is highly variable. Sleep quality also deteriorates with advancing age, as do other circadian rhythms (32); thus, more of our ageing population is likely to experience circadian rhythm disruption. Furthermore, with senescence comes a predisposition to various diseases also characterized by circadian system disorganization, one of which is Alzheimer's disease (33).

Together, it appears that the pervasiveness of circadian rhythm and sleep disruption is rising, and observational evidence implicates this disruption in adverse health effects. Our understanding of the mechanisms underlying these consequences provides the foundation from which to intervene appropriately, and disorganization of the circadian system is at the center of many of these health problems.

## II. The Circadian System

### A. The suprachiasmatic nuclei

**Figure 1.**
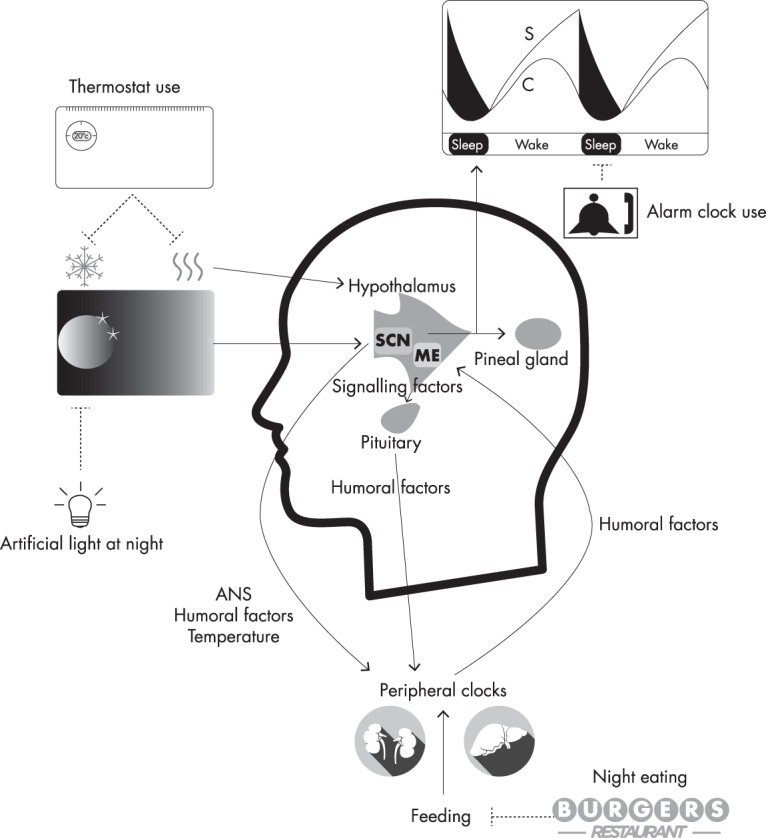
Temporal control of physiology. Light exposure provides the primary time cue for the central clock in the suprachiasmatic nuclei (SCN) of the hypothalamus and suppresses melatonin synthesis by the pineal gland. Artificial light exposure at night can therefore disrupt the SCN clock and melatonin rhythm. As a diurnal species, melatonin is hypnogenic in humans, although a recent prospective study of pinealectomy demonstrated that endogenous melatonin may not have a strong regulatory role in sleep (42). The sleep/wake cycle has been effectively simulated by a two-process model in which a circadian process (C) influences wakefulness and interacts with a sleep-promoting process (S) that accumulates during wakefulness. Within the hypothalamus—a nodal point of body temperature regulation—the SCN influences the circadian rhythm of body temperature, a key synchronizer of clocks in peripheral tissues. The use of thermostats can obviate daily oscillations in temperature, which could perhaps influence some circadian rhythms. In addition to temperature mechanisms, the SCN influences clocks in peripheral tissues through neural signals communicated via the autonomic nervous system (ANS), as well as the timely secretion of signaling factors such as prokineticin 2. Hypothalamic-pituitary-peripheral organ axes are important to hormonal regulation of the circadian system. For example, corticotropin-releasing hormone (CRH) enters the portal system through the median eminence (ME) of the hypothalamus and stimulates the secretion of adrenocorticotropic hormone (ACTH) by the anterior pituitary gland. ACTH then regulates adrenal cortex production of cortisol, a hormone with a robust circadian oscillation and important synchronizing effects in many peripheral clocks. The timing of metabolic processes in peripheral clocks is also modified by nutritional status, and peripheral clocks relay metabolic information back to the hypothalamus through the ME. Today, around-the-clock access to food can distort the clear feeding/fasting cycles that typified much of our history.

The two suprachiasmatic nuclei (SCN) of the anterior hypothalamus primarily coordinate the oscillator systems that temporally regulate physiology and behavior (Figure 1). The preeminent roles of the SCN in locomotor, hormonal, and feeding circadian rhythms were demonstrated by early ablation studies (34–36). SCN transplantation into SCN-ablated hamsters confirmed their control of circadian rhythm period (37) and by encasing the SCN in semipermeable membranes that prevented synaptogenesis, the SCN were shown to produce diffusible signals sufficient to restore circadian activity rhythms (38). Several of these SCN secretions have since been identified (39–41).

The foremost zeitgeber that entrains the SCN to the 24-hour day is the LD cycle, and together with rod and cone photoreceptor cells, melanopsin-containing intrinsically photosensitive retinal ganglion cells in the inner retinae relay photic information to the SCN via a monosynaptic pathway to permit synchronization (43). In response to photic stimuli, a multisynaptic pathway from the SCN to adrenergic fibers innervating the pineal gland regulates norepinephrine release from these fibers and hence melatonin synthesis (44, 45). Melatonin conveys photoperiodic information to the pituitary *pars tuberalis*, a key nexus in the regulation of seasonal rhythms in physiology in photoperiodic mammals, which undergoes seasonal body mass and reproductive changes (46, 47). Although the human melatonin rhythm is also sensitive to photoperiod changes (48), the presence of artificial lighting suppresses seasonal changes in circadian rhythms (such as melatonin) that might otherwise be evident (49). Because the SCN also have the melatonin receptors MT_1_ and MT_2_ (50, 51) (MTNR1A and MTNR1B in humans, respectively), melatonin feeds back to the master clock.

The circadian system has central roles in sleep/wake cycle regulation, as seen by the gating of sleep at specific circadian phases; furthermore, the circadian phase at which sleep occurs influences sleep duration, continuity, and architecture (52). In 1982, Borbély (53) proposed a two-process model of sleep regulation in which a circadian process also interacts with a homeostatic process to regulate sleep. The circadian process influences alertness, and the sleep process is hypnogenic, rising during wakefulness and falling during sleep in a manner akin to an hourglass timer. Borbély's model has proven effective in simulating sleep in myriad experimental conditions (54), but many of the mechanisms by which the circadian system and sleep/wake states interact remain elusive.

### B. Clock genes

Immediate early genes in the SCN respond to light exposure, including clock genes (55), the genes that generate approximately 24-hour gene transcription rhythms. These same clock genes exist in cells throughout bodily tissues and form delayed, interlocking gene transcription/translation negative feedback loops. The positive arm of the core clock loop comprises the basic helix-loop-helix transcription factors circadian locomotor output cycles kaput (CLOCK) and brain and muscle aryl hydrocarbon receptor nuclear translocator-like 1 (BMAL1). In tissues such as the vasculature, CLOCK's functions are replaced by its paralogue neuronal period-aryl hydrocarbon receptor nuclear translocator single-minded protein 2 (NPAS2). Contrary to prior findings, it was recently shown that loss of CLOCK does not produce arrhythmicity in peripheral cells; rather, it appears that these cells only become arrhythmic when *Npas2* is knocked down in the presence of CLOCK deficiency (56), indicating a more prominent role of NPAS2 in the molecular clock than previously thought. CLOCK and BMAL1 heterodimerize to activate transcription of clock-controlled genes (CCGs). CCGs include the negative limb of the clock, namely cryptochrome (CRY) 1–2 and period (PER) 1–3 proteins. These then accumulate in the cytosol, multimerize, translocate into the nucleus, repress CLOCK-BMAL1 transactivating function, and terminate *CRY1–2* and *PER1–3* transcription. PER-CRY complexes are then degraded by casein kinase 1 (CK1) ϵ, CK1δ, and F-box/LRR-repeat protein 3. CLOCK-BMAL1 inhibition ends, thus closing the negative feedback loop. At least five auxiliary feedback loops add robustness and couple the molecular clock to metabolic status (57). Antiphasic to the core loop, the best characterized of these modulates *BMAL1* transcription: RAR-related orphan receptor α activates *BMAL1* transcription, and reverse-erythroblastosis (REV-ERB) α and REV-ERBβ repress *BMAL1* transcription.

The rhythmic transcription of clock genes persists even in cultured fibroblast cells (58), and because fibroblast gene expression periods may be consistent with whole-body circadian rhythm periods (59), clock genes may be key determinants of circadian period and hence chronotype and sleep phenotypes. This is supported by recent genome-wide association (GWA) studies that have linked genetic loci near established components of the molecular clock with chronotype (60, 61). Nevertheless, not all studies have found that in vitro fibroblast period duration is correlated with in vivo period (62). In adulthood, circadian period advances with increasing age, but a difference in in vitro fibroblast period has not always been found between young and elderly individuals. In the presence of sera from the elderly participants, however, fibroblast period was reduced in comparison to treatment with sera from the young adults, suggesting that circulating factors are also determinants of oscillator periods and perhaps chronotype (63). Interestingly, although sleep timing advances with age in adulthood, changes in body temperature rhythm periods are not so clear (64, 65). Because the body temperature rhythm is partly regulated by the SCN, it is plausible that humoral factors influence circadian oscillations in some peripheral cells but not the SCN, although this hypothesis requires testing.

Clock genes regulate the transcription of CCGs, hundreds of which control the timing of tissue-specific functions (66). An aggregation of mouse studies has shown that >50% of protein-coding genes have 24-hour gene expression profiles in at least one set of conditions (67). Although some genes may be rhythmic in multiple tissues, their phases often differ between and even within tissues, and rhythmic gene expression is largely organ-specific (68, 69). Our understanding of the range of healthy phase relationships between these systems is poorly characterized, however.

Target genes of circadian clock genes are enriched for metabolic pathways, and metabolic genes that are direct targets of CLOCK-BMAL1 heterodimer also feedback to molecular clock components. These metabolic genes include D-box binding PAR bZIP transcription factor, differentiated embryo-chondrocyte expressed genes 1 and 2, estrogen-related receptor α, nicotinamide phosphoribosyltransferase, peroxisome proliferator-activated receptor α, and proper homeobox 1 (57).

It is important to note that because the metabolic state of the cell is coupled to the molecular clock (70), the pervasiveness of rhythmic cellular activities is modified by factors such as diet. The circadian system reciprocally interacts with feeding/fasting cycles via coupling between the molecular clock and metabolic regulators. In the postabsorptive state, decreased energy availability increases 5′AMP-activated protein kinase (AMPK) phosphorylation to enhance ATP formation; in the postprandial state, increased energy availability stimulates anabolic cellular processes via mechanistic target of rapamycin signaling. This pathway is coupled to the molecular clock by phosphorylation of glycogen synthase kinase 3β, which in turn regulates PER stability, and hence period length (71). Using AMPK to further exemplify how energy sensors impinge on the molecular clock, AMPK interacts with the NAD(+)-dependent protein deacetylase SIRTUIN (SIRT) 1, which subsequently deacetylates and degrades PER2 to ensure high amplitude daily transcription profiles of several clock genes (72). Furthermore, SIRT1 and SIRT6 have particularly pivotal roles in the temporal control of metabolism by controlling chromatin modifications and hence the rhythmic transcription of distinct sets of genes in the liver, with SIRT1 primarily regulating genes involved in peptide and cofactor metabolism, and SIRT6 influencing genes integral to carbohydrate and lipid metabolism (73).

Finally, although there is contention regarding whether humans are seasonally photoperiodic (74), daily gene expression rhythms occur within the context of seasonal changes in expression of protein-coding genes. Seasonal gene expression changes have been shown in independent populations and are linked to changes in the cellular composition of blood. Seasonal gene expression fluctuations may underlie changes in immune function, and seasonal variations in expression profiles of metabolic genes in adipocytes are also apparent (75). Because the incidences of some cardiometabolic diseases oscillate seasonally (76, 77), these findings may have implications for understanding and treating such pathologies; however, seasonal changes in disease risk may also be related to myriad other factors, including changes in health behaviors and environmental temperature.

### C. Post-transcriptional clock regulation

Within a species, similar proportions of the transcriptome, proteome and metabolome oscillate with 24 hour profiles (78–80). Although there are mostly minimal delays between gene transcription and translation, it has been shown using a human cell model that some arrhythmic gene transcripts produce rhythmic products via daily translation profiles (81). Delays between gene transcription and translation vary across the day, partly due to RNA-binding proteins which modify processes such as pre-mRNA splicing, polyadenylation and RNA decay (82, 83). Post-translational clock protein modifications include acetylation, O-GlcNAcylation, poly-ADP ribosylation, phosphorylation, SUMOylation, and ubiquitination (84–89). Non-coding RNA expression also fluctuates in similar proportions to protein-coding gene transcripts, conferring another level of post-transcriptional regulation, and therefore non-coding RNAs likely influence molecular clock regulation (78). Collectively, such post-transcriptional processes contribute to appropriate, tissue-specific responses of peripheral clocks.

### D. Non-transcriptional oscillators

Non-transcriptional oscillations in peroxiredoxins, redox-sensitive antioxidant proteins involved in electron transfer, respond to oxidation in cells such as erythrocytes (90). These oscillations persist in the absence of zeitgebers, and are temperature-compensated and entrainable. They are sustained in the absence of clock gene expression feedback loops and are the most highly conserved clocks known (91, 92). Their integration with the circadian system, sleep homeostasis, and metabolic networks is little understood, however.

### E. Internal synchrony

Clock gene expression rhythms have divergent periods that do not resonate without synchronizing agents (93). Disruption of the SCN clock dampens and desynchronizes peripheral tissue circadian rhythms (94), and uncoupling of appropriate phase relationships between endogenous rhythms (internal desynchronization) is hypothesized to contribute to the deleterious metabolic effects of circadian rhythm disruption. The SCN synchronize circadian rhythms by autonomic, behavioral, humoral, and temperature mechanisms. The former comprise caudal efferents to the subparaventricular zone and dorsomedial nucleus, dorsal efferents to the midline thalamic nuclei, and rostral efferents to the anterior hypothalamus and preoptic area (95). The paraventricular nucleus is particularly important in the regulation of circadian rhythms in activity, autonomic processes, and secretion of hormones including melatonin and cortisol (96).

Multisynaptic efferents from the SCN to the periphery help regulate the availability of nutrients like glucose in the blood (97), as well as hormone secretion rhythms by organs including the adrenal glands, adipose tissue, pancreas, and thyroid gland (98–101). In turn, humoral factors from the periphery relay information back to hypothalamic regions via the hypothalamic median eminence (102).

Some clock genes are directly regulated by glucocorticoids via glucocorticoid response elements (103). SCN lesions nullify liver gene transcription rhythms, but glucocorticoid receptor activation restores approximately 60% of these rhythms, demonstrating an important synchronizing role for cortisol in some peripheral clocks (104). The strength of effects of glucocorticoids on peripheral clock rhythms differs between tissues; the kidneys and lungs, for example, are yet more responsive to glucocorticoids than the liver, which is more entrained by feeding (105).

As homeothermic vertebrates, humans are resistant to temperature entrainment by the environment, and experiments in mice tissues have shown that the SCN are responsible for this resistance (106). Although thermoregulation is regulated by the interaction of many structures located primarily in the hypothalamus, brainstem, and spinal cord, the SCN are integral to the core body temperature rhythm, a rhythm that has important synchronizing effects on oscillators throughout the body (106). Furthermore, a specific component of the molecular clock has been identified as a key connection between the circadian and thermoregulatory systems because deletion of *Rev-erb*α in mice abolishes the core body temperature rhythm by changing brown adipose tissue activity (107).

### F. Temporal partitioning of physiology

Internal synchrony temporally partitions physiology to aid physical activity and energy harvesting during the biological day (active phase), inactivity and energy mobilization during the rest phase, and time-of-day appropriate changes in immune function (108, 109). Clock-regulated changes in blood pressure, heart rate, and skeletal and heart muscle contractile efficiency and substrate oxidation ready the body for physical activity (110–112). As a result, the circadian system ensures peak physical performance during the active phase.

Historically, physical activity has been necessary to procure food, and during the active phase, rhythms in the gastrointestinal system support timely digestion. Gastric emptying and colonic motility, for example, are slowest in the evening, and the transit of indigestible food from the stomach to the small intestine is powered by the migrating motor complex, the speed of which is more than twice as high during the day as at night (113–115). Rhythmic bile acid production is central to cholesterol metabolism and absorption of nutrients, including fat-soluble vitamins. Kruppel-like factor 15 and fibroblast growth factor 15 have pivotal roles in this regulation (116). Rhythmic changes in the activity of some intestinal nutrient transport proteins (117) and temporal control of enzyme activity are also important to metabolism. Using lipids to exemplify this, reduced postprandial lipoprotein lipase activity apparently contributes to impaired lipid tolerance in the evening (118). It should be noted that it is unclear whether the aforementioned gastrointestinal rhythms are partly clock-regulated or exclusively artifacts of behavioral cycles. Other enzymes involved in hepatic lipid metabolism are known targets of the molecular clock and shape rhythms in processes like lipid accumulation (119). Nocturnin, for example, is clock-controlled and regulates triacylglycerol synthesis and storage, as well as chylomicron formation in intestinal enterocytes (120).

Gut microbiota composition changes with feeding and fulfils time of day-specific functions in humans and mice, with energy metabolism roles during the active phase and detoxification processes during the rest phase (121). Reciprocity between the circadian system and microbiota is evident because *Bmal1* deletion nullifies these oscillations (122). Furthermore, clock gene expression is altered in germ-free mice, and these mice do not gain body mass compared to conventionally raised mice, perhaps due to differences in microbe-derived metabolites like short-chain fatty acids (123).

Whereas it is unclear whether rhythmic secretion of several gastrointestinal hormones is shaped by the circadian system (such as gastric inhibitory polypeptide, gastrin, and glucagon-like peptide-1) (124, 125), other hormones are demonstrably clock-regulated. Constant routine and forced desynchrony experiments have shown that plasma glucose and triacylglycerol have clear circadian rhythms, with an acrophase in the biological night (126–128), indicating circadian system regulation of energy substrate metabolism. This is likely related to circadian rhythms in important hormones in energy metabolism, such as insulin. Insulinemia, like insulin sensitivity, peaks in the daytime in humans to promote efficient energy storage (128), and an acrophase in the insulin-sensitizing adipokine adiponectin in the middle of the active phase supports this process (129).

Rhythmic growth hormone (GH) secretion is another important determinant of daily changes in substrate oxidation. Although a minor influence of the circadian system has sometimes been detected, sleep has a dominant influence on GH secretion (5). GH has an acrophase near the onset of slow wave sleep (SWS), and GH profiles are further characterized by episodic surges a few hours after meals (130, 131). The GH rhythm differs a little between the sexes (132). Together with insulin and insulin-like growth factor 1 (IGF-1), GH aids nitrogen retention during high energy availability; during reduced energy availability, as occurs during sleep, GH stimulates lipolysis and ketogenesis (133), inducing insulin resistance and thereby sparing glucose and protein oxidation (134).

Like GH, prolactin is primarily synthesized and secreted by the anterior pituitary and fulfils >300 biological actions, including roles in homeostasis, lactation, and reproduction (135). Prolactin also influences appetite in a species-specific way, and chronic hyperprolactinemia is associated with increased body mass in humans. Furthermore, prolactin has roles in lipid metabolism, largely reducing lipid storage in adipocytes, and also affects glucose metabolism by stimulating insulin secretion (136). A circadian rhythm in prolactin is evident in constant routine protocols, of larger amplitude in women and with an acrophase in the rest phase (137). Subcutaneous prolactin injections prolong rapid eye movement sleep in rats (138), and prolactin is also associated with SWS in humans (139).

Melatonin synthesis occurs during darkness and increases sleep propensity in humans, which may be related to melatonin's hypothermic effects (140). Melatonin may have important roles in metabolic regulation, perhaps helping prevent nocturnal hypoglycemia by inhibiting insulin secretion (141). Because this was demonstrated in mice, however, different responses may be apparent in humans. Interestingly, the *MTNR1B* T2DM risk variant rs10830963 (142–144) has been linked to prolonged melatonin synthesis duration and delayed melatonin offset phase in humans. Because melatonin inhibits glucose-stimulated insulin secretion ex vivo, it is plausible that extended melatonin synthesis into waking could contribute to T2DM risk, particularly among carriers with early sleep times (145). As melatonin also exerts receptor-independent effects in free radical scavenging (146), melatonin perhaps also contributes to temporal regulation of immune function, although further research is required.

Finally, an acrophase in leptin secretion during the rest phase may contribute to reduced appetite for most foodstuffs in the biological morning in humans, permitting consolidated sleep despite declining energy availability (147). This hypothesis is not supported by the finding that nocturnal rats also have an acrophase in leptin secretion during darkness (their active phase) (148), although it is possible that leptin has different roles in diurnal and nocturnal species.

## III. Consequences of Circadian Rhythm and Sleep Disruption

### A. Sleep restriction and sleep deprivation

#### 1. Metabolic consequences

**Figure 2.**
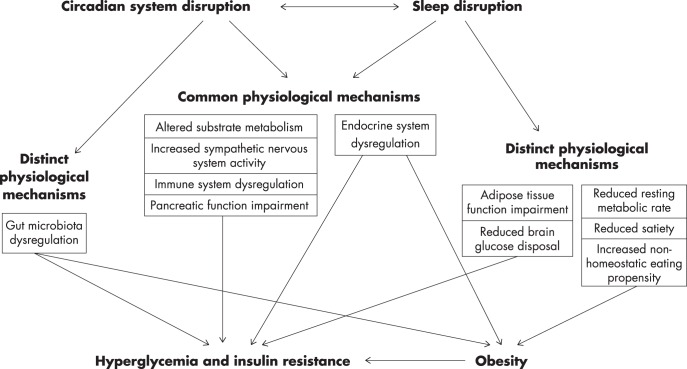
Mechanisms linking circadian system and sleep disruption to hyperglycemia, insulin resistance, and obesity. With further research, mechanisms that are currently listed as distinct may prove to be common.

Sleep restriction is ubiquitous, and its metabolic consequences are profound. One of the best-characterized metabolic sequelae of sleep disruption is disrupted glucose metabolism. This was first shown in 1999 in a study in which participants experienced a 24% reduction in insulin sensitivity after five nights of sleep restriction to 4 hours per night. Altered 24-hour endocrine profiles were also evident because TSH secretion was impaired and nocturnal cortisol secretion increased (149). This finding of abnormal glucose metabolism has been consistently replicated, and much progress has been made in understanding the contributing mechanisms (Figure 2).

In the postprandial state, the brain accounts for roughly half of whole-body glucose disposal, and sleep deprivation reduces this use (150). Cephalic phase neurogenic signals from the brain anticipate food consumption and stimulate insulin secretion. Insulin resistance after sleep restriction is not associated with altered cephalic phase insulin secretion (151) but rather appears to result primarily from insulin resistance outside the liver (152), and changes in adipose tissue insulin signaling may be particularly important (153). Given the roles of the molecular clock in glucose and lipid metabolism, reduced glucose tolerance and metabolic dysregulation after sleep disruption may also be related to epigenetic and transcriptional changes in the molecular clock in peripheral tissues important to glucose disposal, including adipose tissue and skeletal muscle. Indeed, there is increased DNA methylation of the promoter region of *CRY1* and two regions near *PER1* in adipocytes, as well as reduced *BMAL1* and *CRY1* transcription in myocytes after sleep deprivation (154). Consistent with Randle's glucose fatty-acid cycle (155), increased release of nonesterified fatty acids from adipocytes after sleep restriction likely also contributes to insulin resistance (156). Further mechanisms reducing insulin sensitivity after sleep disruption include stimulation of gluconeogenesis via increased sympathetic activity of the autonomic nervous system (157) and a shift in cytokine balance toward a more inflammatory state (158). Related to this, sleep curtailment affects numerous aspects of immune function. For example, 1 week of sleep restriction in men increased circulating white blood cells and changed their diurnal rhythm. Notably, altered cell counts had not returned to baseline after 9 days of recovery sleep (159). Such changes in immune function may contribute to the development of diseases associated with immune system changes, such as T2DM (160).

It has also been shown that changes in sleep architecture during sleep disruption contribute to changes in glucose metabolism. Indeed, restricting sleep to the first half of the night produces distinct endocrine effects compared to restriction to the second half (161), and different sleep stages produce distinct physiological changes. Selective SWS restriction, for example, reduces insulin sensitivity in a dose-response manner in adults, independent of sleep duration, although adolescents may be more resistant to this effect (162, 163).

Many individuals, especially late chronotypes, use alarm clocks to artificially curtail sleep during the work week; simulating this behavior by enforcing 5 days of restriction to 5 hours of sleep with early waking during the rest phase reduced intravenous and oral insulin sensitivity by approximately 20% in healthy adults (164). Intravenous insulin sensitivity was not restored by 3 days of recovery sleep; notably, 3 days is longer than most working adults have to catch up on sleep each week. A limitation of most experimental sleep restriction studies is that they often enforce large changes in sleep duration, but restriction by 90 minutes per night—an amount similar to that experienced by many (18)—has also been shown to reduce insulin sensitivity after 1 week in young men (165). In this healthy population, impaired insulin sensitivity dissipated with continued exposure to such restriction.

A detailed review of obstructive sleep apnea (OSA) is beyond the scope of this review. However, because studies have consistently shown that OSA is associated with impaired insulin and glucose metabolism, its features are briefly outlined. OSA is a disorder in which individuals experience episodic upper airway closure and hence intermittent hypoxia during sleep. OSA is further characterized by reduced sleep duration, sleep fragmentation, reduced SWS, and increased sympathetic nervous system activity and oxidative stress, all of which can contribute to insulin resistance (166). Obese individuals are at particular risk of OSA, and its prevalence has risen in recent years in the United States, with estimates suggesting that 10% of 30- to 49-year-old men, 17% of 50- to 70-year-old men, 3% of 30- to 49-year-old women, and 9% of 50- to 70-year-old women experience OSA (167). Although obesity increases the risk of OSA and is itself associated with insulin resistance, insulin resistance in OSA has been shown to be independent of adiposity (168). Furthermore, OSA is highly prevalent in people with T2DM (169), and a meta-analysis has shown that OSA is a strong risk factor for T2DM development (170). Pregnancy also predisposes women to OSA (171) and may worsen maternal glucose metabolism and hence increase the risk of gestational diabetes (172). Continuous positive airway pressure is used to treat OSA; whether it improves glucose metabolism is contentious, but accumulating evidence suggests that it may (173). We would also be remiss if we did not mention that the metabolic consequences of insomnia have not been thoroughly studied, despite it being the most common sleep disorder. Because sleep state misperception (the mistaken perception of wakefulness during sleep) is common in insomnia, it will be important to measure sleep objectively in these studies.

Although conflicting evidence exists (174), a meta-analysis of sleep restriction studies showed that sleep restriction increases energy intakes in adults, contributing to its obesogenic effects (175). This finding is consistent with an extended period in which food can be consumed to compensate for the additional energetic cost of wakefulness. Sleep restriction also increases energy intakes and the appeal and consumption of desserts among adolescents (176), and sleep deprivation has even been shown to increase the energy content and mass of food purchased per unit of money in a mock supermarket (177).

In light of these findings, it might be expected that sleep restriction influences satiety hormones, of which the best-studied are ghrelin and leptin. During ad libitum food availability, however, documented effects of sleep restriction on ghrelin and leptin are contradictory (149, 178, 179). With that said, sleep restriction does appear to increase ghrelin concentrations and hunger in adults consuming standardized hypoenergetic diets (180) and after sleep restriction, ghrelin is subsequently positively correlated with energy intake when eating ad libitum (181). It is important to consider that a multitude of hormones influence feeding behavior, and it was recently shown that sleep restriction increases plasma concentrations of the orexigenic endocannabinoids 2-arachidonoylglycerol and 2-oleoylglycerol, perhaps also contributing to increased energy intakes (182).

The energy expenditure side of energy balance may also be affected by sleep restriction, as a working week sleep restriction simulation study showed that sleep restriction reduces resting metabolic rate (RMR), particularly among African Americans—a population highly susceptible to the obesogenic effects of sleep restriction. Notably, this effect was seen despite participants being in positive energy balance, which might be expected to have raised their RMRs (183). Lean body mass is a key determinant of RMR, and sleep restriction during hypoenergetic diet consumption accelerates lean body mass losses and impairs reductions in adiposity, providing a mechanism by which chronic sleep disruption could detrimentally influence body composition (184). The importance of adequate sleep during energy restriction is supported by the finding that longer sleep predicts greater reductions in BMI in overweight and obese adults consuming hypoenergetic diets (185).

Interestingly, within-participant effects of one night of sleep restriction on energy intake and body mass changes appear stable when repeated exposures are separated by long periods of time, particularly among men, suggesting trait-like responses to sleep disruption. Given large differences between individuals in changes in body mass (−2.3 to +6.5 kg) and energy intakes (−501 to +1178 kcal) after one night of sleep restriction (186), and also given that many sleep variables are highly heritable (187), there is a need to find biomarkers that identify those most vulnerable to adverse metabolic effects. Some gene variants, like the Y362H variant of basic helix-loop helix family member e41, have been shown to confer carriers with resistance to other effects of sleep deprivation, such as less need for recovery sleep (188); perhaps biomarkers that influence resistance to the metabolic sequelae of sleep disruption will also be identified. The use of “omics” technologies to study sleep disruption is a particularly promising way of revealing mechanisms underlying this interindividual variability.

Tremendous advances have been made in various omics techniques in recent years, including genomics, transcriptomics, proteomics, and metabolomics. Their high throughput has particularly promising applications in studying the circadian system and sleep, as exemplified by the characterization of metabolites affected by sleep deprivation by the use of liquid chromatography/mass spectrometry metabolomics, a method that can also be applied in field settings (189).

Studies applying metabolomics, proteomics, and transcriptomics continue to unveil new insights into sleep and the circadian regulation of metabolism. Circadian system regulation of physiology is reflected in the human metabolome, approximately 15% of which is clock-regulated. Under constant routine conditions, approximately 75% of oscillating blood plasma metabolites are lipids, generally with acrophases around lunchtime (190). A targeted lipidomics study has shown that approximately 13% of plasma lipids show 24-hour oscillations, including lipids involved in energy storage, signaling, and transport. Interestingly, there is large heterogeneity among healthy adults, and different lipid metabolic phenotypes have been identified (191). In saliva, amino acids and associated metabolites comprise over half of the oscillating metabolites (190). When entrained to the LD cycle, the proportion of rhythmic metabolites may be higher still, with 64% of measured plasma metabolites found to have 24-hour rhythms, 87% of which peaked during the day (189).

Omics techniques have also been applied to the study of circadian rhythm and sleep disruption. Sleep restriction primarily modifies lipid, neurotransmitter, oxidative stress, and gut metabolites (192), effects that may help explain increased cardiometabolic disease risk in sleep-restricted individuals (193). Similarly, sleep deprivation alters rhythms in plasma metabolites including lipids and acylcarnitines, largely reducing the amplitude of metabolite rhythms in comparison to when sleep is permitted (189). Discoveries using transcriptomics corroborate many metabolomics findings: 1 week of sleep restriction in humans reduces the number of genes with 24-hour expression profiles by >20% in the blood, influencing genes involved in immunity, gene expression regulation, stress responses, and metabolism (194). The extent of these changes can be explained by the diversity of changes that occur during sleep, including changes in physical activity, light exposure, redox state, and temperature, all of which influence numerous physiological processes. Furthermore, complete (180°) circadian misalignment profoundly affects the human blood transcriptome, producing a 6-fold reduction in genes with 24-hour expression profiles, an effect consistent with reduced core body temperature rhythm amplitudes (195).

The use of multiomics techniques has the potential to reveal novel insights into the systems-level regulation and integration of the circadian system and sleep homeostasis, in addition to identifying novel biomarkers of metabolic dysfunction and circadian system and sleep disruption. Online repositories for omics datasets (for example, Circadi0mics; http://circadiomics.igb.uci.edu/) could facilitate discoveries by integrating multiple omics approaches and displaying temporal aspects of verified and predicted network interactions between key metabolic regulators, such as enzymes and transcription factors (196).

There are conflicting findings regarding whether men and women differ in their energy balance responses to sleep disruption. A large study of five nights of sleep restriction (4 hours of time in bed) found that men are predisposed to positive energy balances after sleep restriction (197); however, a smaller, crossover study of five nights of sleep restriction (5 hours of time in bed) in a more homogeneous group of younger adults indicated that women are more susceptible (198). Further research is required to understand these discrepant findings. Certainly, sex does influence certain responses to sleep restriction, as seen in menstrual cycle phase-dependent endocrine responses to sleep restriction (199), and there is a general need to clarify how the menstrual cycle influences responses to circadian rhythm and sleep disruption.

Finally, it is likely that changes in sleep architecture during sleep disruption influence eating behavior: The final rapid eye movement period, for example, is hypothesized to be protective against overeating (200). Experiments should continue to assess associations between sleep architecture, behavior, and physiology.

#### 2. Effects on dietary choices

It has been estimated that U.S. adults make approximately 230 food-related decisions daily (201). If sleep disruption adversely influences dietary choices, its deleterious metabolic effects could be compounded. Although sleep restriction has sometimes been found to influence dietary macronutrient proportions, conflicting evidence exists (197). Notably, macronutrient intakes depend on available foods, and limited snack options are available in experimental settings. Rather than altering macronutrient preferences, recent brain imaging studies support the hypothesis that sleep disruption increases nonhomeostatic eating propensity.

Sleep restriction accentuates increased activity in brain regions involved in reward in response to food stimuli (202), suggesting heightened sensitivity to rewarding properties of food. Brain activity changes after sleep deprivation are consistent with increased appetite (203), and activity in one of these regions, the nucleus accumbens, is particularly highly associated with energy-dense food selection (204). Furthermore, sleep restriction strongly influences insula activation in response to images of food perceived as “unhealthy,” a region involved in pleasure seeking, even after a day in which sleep-restricted participants consumed more energy than control participants (205).

Finally, accurate recollection of food consumption influences short-term food ingestion, an extreme example of which is seen in amnesiacs who will eat multiple meals consecutively (206). Hippocampal changes after sleep deprivation contribute to memory impairments (207), and hippocampal activity helps prevent meal initiation in the postprandial period (208). Although it is plausible that sleep disruption may increase food intake by influencing hippocampal activity, this hypothesis requires further testing.

Collectively, these studies suggest that experimental sleep restriction and deprivation induce a plethora of adverse metabolic consequences that may be accentuated by changes in food selection. Experimental sleep disruption is often more severe than that experienced outside the laboratory; therefore, studies of less marked sleep restriction and further field studies are needed.

### B. Limited daytime light exposure

People in industrialized societies typically spend approximately 88% of their time in enclosed buildings, sheltered from natural light (209). Time spent outdoors in Canada, Great Britain, and the United States is commonly 1 to 3 hours daily (210–212), depending on season and other factors, and compared to exposure to only natural light, individuals in modern societies are perhaps exposed to about four times less light during the day (213). Consequently, many individuals, particularly those in urban populations, are sheltered from the diverse beneficial effects of natural daytime light on behavior and physiology (214).

Vitamin D is synthesized in response to UV-B irradiation, and indoor living in industrialized areas is one contributor to low vitamin D status. Although consistent evidence linking vitamin D status to morbidity is contentious, associations with some health parameters exist (215). It is well established that the LD cycle is the primary zeitgeber for humans; however, because vitamin D directly influences clock gene transcription in vitro (216), it is possible that some of the associations between vitamin D status and health may arise from effects of vitamin D status on circadian rhythms and sleep. Negative associations between vitamin D status and sleep duration (217–219) and sleep efficiency (217, 219) corroborate this contention; nevertheless, this may simply reflect beneficial effects of greater daytime light exposure on sleep rather than effects of vitamin D on the molecular clock.

### C. Increased light exposure at night

Although outdoor light levels do not always reflect retinal light exposure, about 75% of the world's population is exposed to artificial light at night (220), and it has been estimated that individuals in modern societies commonly experience light intensity levels over twice as high between sunset and sleep compared to exposure to only natural light (213). This appears to significantly influence sleep. The introduction of electric lighting is associated with increased light exposure shortly after dusk during workdays, delayed sleep onset, and shortened sleep duration among individuals of the same sociocultural background. These effects may be particularly prominent during workdays (221, 222). Although the authors of a recent study of three hunter-gatherer communities living without electricity suggested that individuals living without artificial lighting may not sleep for longer than individuals with lighting (223), this suggestion was not supported by a comparison between the hunter-gatherer groups and individuals of similar ethnic origins with access to artificial lighting (224).

Many electronic devices also now increase nighttime light exposure. Given their compactness, it might be expected that any effects of these devices on the circadian system and sleep would be benign. However, some of these devices emit monochromatic blue light (λ_max_, 460–480 nm), to which intrinsically photosensitive retinal ganglion cells are especially sensitive. Indeed, irradiance levels as low as 2 μW/cm^2^ of such light suppress nocturnal melatonin production (225, 226). As a result, nighttime exposure to even low levels of light from e-Book devices delays sleep and dim-light melatonin onset, reduces melatonin synthesis, and impairs next-morning alertness. Because about 90% of Americans use electronic devices within an hour of bedtime on multiple nights each week, these devices are likely further contributing to circadian rhythm and sleep disruption (227).

There are positive associations between nighttime illumination and obesity prevalence in more than 80 countries worldwide (228), and also between the mean timing of light exposure above 500 lux and BMI in free-living adults (229). Given the aforementioned discussion of the sleep-disrupting effects of light exposure, as well as the multitude of factors that conspire to increase energy balance after sleep disruption, it seems likely that increased light exposure at night is another contributor to the obesity epidemic.

### D. Shift work

Even when diet is controlled, night shift workers exhibit poorer metabolic health than day workers. Night workers, for example, have higher plasma triacylglycerol (230), and field work has shown that this is related to circadian system disruption because postprandial glucose and lipid tolerance to standard test meals are impaired on switching to night shifts (231). Like metabolic health, cognitive function is frequently impaired by night shift work. Transitioning from day to night shifts often entails sleep deprivation, and performance decrements during this time can be comparable to blood alcohol levels that exceed the legal driving limit (232).

An important determinant of shift work tolerance is entrainment to shift schedules. Isolated environments can be more conducive to adaptation to shift work than more common shift working scenarios, and in circumstances such as workers experience on the British Antarctic base at Halley or on oil rigs, most workers can synchronize their circadian systems to night shifts within a week (233). Even in these instances, however, it can take weeks for workers to re-entrain to day shifts (234–236).

Workers who exclusively work night shifts might be expected to completely entrain their circadian systems to their work hours (6). However, disrupted endocrine circadian rhythms persist even among adults who have worked night shifts for over 2 years. These chronic night shift workers have altered TSH profiles, and reduced cortisol secretion and increased prolactin secretion during their waking hours may impair vigilance (237).

Laboratory experiments have attempted to simulate shift work to clarify its metabolic consequences. As discussed previously, it is apparent that sleep restriction reduces insulin sensitivity; however, circadian misalignment imposed by LD cycle shifts nearly doubles reductions in insulin sensitivity after sleep restriction alone in males, also furthering inflammation (238). Some shift work simulation experiments have produced conflicting findings regarding several metabolic variables. During 6 days of simulated night shift work in healthy adults, there was an initial increase in fat oxidation and a reduction in carbohydrate and protein oxidation. Diet-induced thermogenesis temporarily fell, and energy expenditure declined on the second and third days, particularly during sleep. Paradoxically, appetite diminished despite reduced levels of the orexigenic hormones leptin and peptide tyrosine tyrosine (239). However, in contrast to these findings, neither three consecutive 3-hour LD cycle advances nor three consecutive 3-hour LD cycle delays influenced appetite or energy expenditure in adults, and both shifts increased carbohydrate oxidation and reduced protein oxidation. In this study, LD cycle advances acutely reduced cortisol rhythm amplitudes and increased insulinemia, whereas LD cycle delays increased glycemia, and decreased glucagon-like peptide-1 concentrations and sleeping energy expenditure (240). Discrepancies between some of these findings likely reflect differences in experimental design, emphasizing the need to compare a variety of shift schedules when attempting to offset deleterious consequences of shift work.

Other circadian misalignment protocols have provided new insights into the short-term health consequences of shift work. Circadian misalignment increases blood pressure (particularly during sleep) and inflammatory markers, reverses cortisol rhythms, and reduces heart rate variability and insulin sensitivity in healthy adults (128, 241). Furthermore, misalignment increases postprandial glycemia despite enhanced late-phase insulin secretion (242). Prolonged circadian misalignment reduces cortisol secretion (243), and whereas acute circadian misalignment increases insulin secretion (128), 3 weeks of combined sleep restriction and circadian misalignment impairs insulin secretion, indicating pancreatic dysfunction (244). An important question is whether chronic exposure to circadian rhythm and sleep disruption produces adaptations that mitigate the adverse effects of subsequent disruptions to the endocrine system. It appears that this may not be the case, however, because healthy chronic shift workers are still subject to the deleterious effects of circadian misalignment on postprandial glucose tolerance and insulin action (245).

Collectively, these studies indicate possible mechanisms linking shift work to increased metabolic disease risk and show a need to optimize shift work schedules (direction, duration, and frequency) to minimize deleterious health effects. In general, changing from backward to forward shift rotation, shifting from slow to fast shift rotation, and allowing self-scheduling of shifts appear to benefit the health and quality of life of shift workers (246). Superficially minor details can profoundly influence adaptation to shift work, as demonstrated by delayed circadian phase (∼3 hours) and shortened sleep (∼1 hour) in offshore workers whose night shifts were just an hour later (247). Because individual tolerance to shift work varies widely (248), it will be important to find ways of determining those at highest risk of shift work-induced circadian rhythm and sleep disruption. There is a need to study further how to optimize shift work schedules based on chronotype (249) because observational evidence suggests that associations between shift work schedules and T2DM are related to chronotype (250), and manipulating shift work schedules based on chronotype can reduce circadian rhythm and sleep disruption (251). Notably, because shift work and jetlag are both initially characterized by attempts to abruptly change sleep/wake cycle timing, many of the metabolic aberrations seen in acute shift work may be applicable to individuals experiencing jetlag.

## IV. Circadian System Genetics and Metabolism

Because the circadian system is intertwined with metabolic regulation, recent studies have focused on deciphering whether circadian system gene single-nucleotide polymorphisms (SNPs) are associated with metabolic health in adults. Studies of clock gene disruption in other animals paved the way for these studies because whole-body and tissue-specific clock gene mutation and knockout models produce various feeding and metabolic aberrations in rodents (252, 253). Perhaps the most severe example of this is the abolition of behavioral and molecular circadian rhythms in mice after *Bmal1* knockout. These animals also succumb to premature mortality (254).

In rare cases, genetic abnormalities produce developmental disorders that entail circadian system disorganization and metabolic dysfunction. Retinoic acid induced 1 (RAI1) transcriptionally regulates *CLOCK*, and haploinsufficiency of *RAI1* is the primary contributor to the Smith-Magenis syndrome phenotype, a disorder characterized by circadian rhythm and sleep disruption, intellectual disability, and obesity (255).

GWA studies provide the strongest evidence for roles of more common circadian system gene variants in human metabolism and disease risk. GWA studies have linked *PER3* to T2DM (256) and *CRY2* with fasting glycemia and insulin concentrations (257). In these studies, melatonin receptor 1B (*MTNR1B*) variants are also consistently associated with insulin secretion and T2DM risk (142–144).

Less robust evidence for ties between circadian system gene variants and metabolism comes from candidate gene studies. In adults, two *BMAL1* haplotypes have been linked to hypertension and T2DM (258, 259). *CLOCK* SNPs have been associated with nonalcoholic steatohepatitis, metabolic syndrome, small dense low-density lipoprotein levels, obesity, and T2DM (260–264). Perhaps the most studied of these associations is that of obesity; to date, eight common SNPs have been linked to obesity, and three have been associated with energy intakes (265).

An ultimate goal of candidate gene studies is to help personalize healthcare. A recent meta-analysis of up to 28 190 participants from 15 cohort studies sought to identify whether nutrition and sleep modify associations between select circadian system gene variants and cardiometabolic traits. Carbohydrate intake was positively associated with fasting glycemia in the presence of the T allele of *MTNR1B* rs1387153 (266). Moreover, long (>9 hours) sleep was associated with increased BMI in the presence of this allele. Finally, sleep duration was positively associated with high-density lipoprotein cholesterol among carriers of the A allele of *CRY2*-rs11605924. *CLOCK*, *CRY*, and *REV-ERB*α variants were not found to interact with nutrition to influence cardiometabolic health, however.

Collectively, SNP studies suggest that the knowledge of circadian gene SNPs may eventually help identify those at greatest risk of some diseases and personalize interventions. However, candidate gene studies are limited by their sample sizes, their exclusion of all causative genes and gene variants, and their limited replicability. Hence, such studies need replicating in larger, unbiased GWA studies.

## V. Countermeasures Against the Metabolic Consequences of Circadian Rhythm and Sleep Disruption

Several interventions have promise in mitigating the metabolic consequences of circadian rhythm and sleep disruption (Figure 2). It is important to note that circadian rhythm and sleep disruption can have distinct effects, and sleep timing per se has but small effects on the circadian system phase (267); it is perhaps changes in light exposure during sleep because of the closing of the eyes that is likely to have the strongest effect on the circadian system. Nevertheless, the homeostatic regulation of sleep is intertwined with the circadian pacemaker in the SCN, so strategies to counter each should consider this interaction, and interventions should be tailored to individual circumstances.

In the case of sleep restriction, sleep extension appears to benefit many aspects of metabolic health. Among short-sleeping adults, increased time in bed after a sleep extension intervention is associated with improvements in glucose regulation and insulin sensitivity (268), and as little as 3 days of sleep extension may benefit insulin metabolism and increase testosterone in habitually short-sleeping men. Interestingly, sleep extension reduced the anorexigenic peptide hormones leptin and peptide tyrosine tyrosine, although effects on food intake were not assessed (269). Although the need to nap during the daytime may reflect less robust sleep/wake rhythms and hence be associated with adverse health effects including excessive inflammation (270), the use of morning and afternoon napping after sleep restriction reduces increased afternoon urinary norepinephrine excretion toward normal values and also returns salivary IL-6 levels toward baseline from suppression (271).

Sleep extension may also improve body composition. Indeed, increased sleep duration is prospectively associated with attenuated increases in adiposity in short-sleepers (272), and findings from a study of overweight, habitually short-sleeping young adults suggest that this may be related to increased energy expenditure. Among these individuals, 2 weeks of 2-hours of increased time in bed in home environments increased sleep duration and daytime energy expenditure, and reduced appetite and the desire for highly palatable foods (273). One factor that may influence the effects of sleep extension interventions on dietary habits is chronotype. Using a crossover design to change the time at which adolescents went to bed and thereby compare a 6.5-hour to a 10-hour sleep opportunity for five nights, longer sleep opportunity reduced evening eating among individuals with earlier chronotypes only, despite similar sleep timing and duration between chronotypes (274). Hence, there is a need to study further the influence of chronotype on appropriate bedtime recommendations. Finally, sleep extension may benefit other behaviors. A systematic review of naturalistic studies recently found that delaying school start times increases students' sleep durations, and there was some evidence for concomitant improvements in behavior and affect (275). Improvements in sleep hygiene are a natural starting point in attempts to achieve better sleep, and further research on optimizing such variables as sound, bedding, mattresses, and temperature may benefit sleep.

Pharmaceutical chronobiotics have potential in countering circadian rhythm disruption. For example, pharmaceutical inhibition of CK1 helps synchronize misaligned oscillators and hence speeds adaptation to LD cycle shifts in mice (276, 277), as can therapeutic SCN neuropeptide modulation (278). Furthermore, an array of clock-enhancing small molecules may ultimately provide effective therapies for disorders of the circadian system (279). Intriguingly, a circadian clock was recently transplanted into a noncircadian organism for the first time, and such methods could have chronotherapeutic applications, such as regulation of timely drug release (280). This is particularly pertinent given that most of the highest-selling drugs target the products of rhythmically transcribed genes (78). Of note, pharmaceutical chronobiotics may also benefit metabolic health. REV-ERB agonists, for example, diminish adiposity, hyperglycemia, and hyperlipidemia in diet-induced obese mice (281). However, none of these compounds has yet been tested for safety or efficacy in humans.

Other well-established therapies like melatonin (282) should be further studied for effects on food selection and metabolism in humans. Interestingly, 1 year of melatonin supplementation was recently shown to increase lean body mass and reduce fat mass in postmenopausal women with osteopenia (283), and daily ingestion of prolonged-release melatonin reduced glycosylated hemoglobin in individuals with insomnia and T2DM (284). Finally, caffeine, the most commonly consumed psychoactive substance worldwide, changes the period of both the molecular clock in vitro and the melatonin rhythm in humans, and timely caffeine ingestion may therefore help entrainment in such circumstances as jetlag (285, 286).

Nonpharmaceutical interventions are also capable of improving circadian system function and sleep, including blue-blocking glasses and apps to filter short-wavelength emissions from electronic devices (287, 288). Because the brightness, color, duration, and timing of light exposure influence many physiological functions (289–291), it is feasible that these interventions could influence the phase of the SCN and numerous other processes independent of the SCN, including activity in other brain regions and endocrine networks that help regulate appetite. By enabling consumers to have greater control of their light environments, developments in “smart” light technology may be particularly important to improving circadian system alignment and optimizing changes in sleep pressure to coincide with times appropriate for individual chronotypes. In a similar vein, consideration should be given to light exposure when designing buildings and their windows.

The timing, composition, and quantities of foods ingested influence the circadian system (292), as exemplified by the restoration of behavioral and physiological rhythms in SCN-ablated rats by a single, timed, hypoenergetic daily meal (293). Time-of-day-restricted feeding (TRF) limits feeding to a period of several hours and thereby produces clear feeding/fasting cycles. Because the molecular clock is intertwined with the metabolic state of the cellular environment, TRF to the rest phase can invert clock gene rhythms in many peripheral tissues (294). When TRF is imposed with ad libitum food availability in mice, SCN gene expression rhythms are similar to those without TRF, and TRF can therefore change the phase relationships between gene expression rhythms in peripheral tissues and the SCN. Hypoenergetic diets, however, can phase-shift peripheral tissue gene expression profiles and also have a small influence on SCN gene expression rhythms (295). Whether similar effects of TRF schedules are evident in humans is currently unclear; if evident, TRF may too be a useful strategy in situations where resynchronization with the external environment is desired. TRF produces an array of metabolic health benefits in various animals (296), but little research has explored whether this is true of humans. It is apparent, however, that many of us eat in a very erratic manner, and a pilot study of eight overweight adults found that reducing the habitual feeding period from >14 hours to 10–11 hours reduced energy intake and body mass and improved perceptions of sleep (297). Furthermore, there is some evidence that the timing of food intake may influence the effectiveness of weight loss interventions because greater energy intake earlier in the day has been associated with greater weight loss in prospective studies of overweight and obese adults (298, 299).

Finally, reciprocity between physical activity and the circadian system exists because both experimental circadian rhythm disruption and diseases associated with SCN dysfunction disrupt physical activity patterns (300, 301), and physical activity influences melatonin rhythms a little, as well as peripheral tissue gene expression rhythms (302, 303). Furthermore, the circadian system regulates autonomic control of cardiovascular responses to exercise, resulting in peak cardiac vagal tone withdrawal in the morning, and a bimodal acrophase in epinephrine and norepinephrine reactivity to exercise in both the morning and evening, perhaps helping to explain the increased risk of cardiovascular events at these times (304). The extent to which the beneficial metabolic effects of exercise are mediated by the circadian system is unclear; regardless, the health-promoting effects of exercise are extensive. Most human circadian rhythm and sleep disruption experiments to date have enforced minimal physical activity. Rodent studies suggest that physical activity offsets some adverse metabolic effects of light exposure at night (305). Exercise may also offset some of the deleterious effects of sleep disruption because resistance training attenuates the catabolic effects of sleep deprivation on lean body mass in rats, perhaps by offsetting changes in testosterone, IGF-1, and corticosterone (306). Because there is a paucity of human studies on the subject, it will be important to study how to optimize exercise protocols to mitigate metabolic dysfunction induced by circadian rhythm and sleep disruption.

## VI. Conclusions

In modern societies, circadian rhythm and sleep disruption are perhaps more pervasive than ever. There is increasing evidence of detrimental effects on metabolic function and dietary choices, emphasizing the importance of bolstering circadian system function and addressing sleep disruption. Because an appreciation of the importance of circadian system entrainment and sleep may significantly enhance health and productivity for many individuals, educating key personnel has great potential to benefit society.

The circadian system optimizes behavior and physiology according to the time of day and is organized in a hierarchical manner with a central clock in the SCN that is primarily entrained by light. Nowadays, we are commonly exposed to less light during the day and more light at night because of artificial lighting, which may impair circadian system organization and disrupt sleep, resulting in widespread adverse effects on metabolic health. Disrupted sleep, for example, promotes increased energy intake, reduced energy expenditure, and insulin resistance in many individuals, consequences that may be compounded by an increased propensity to make less healthy dietary choices. Careful experiments have also shown that circadian misalignment produces an array of metabolic abnormalities.

Future research should continue to study factors influencing individual variation in the risk of and responses to circadian rhythm and sleep disruption, such as sex differences in circadian rhythms (307), associations between ethnicity and sleep variability (308), and other factors that contribute to differences in metabolic and behavioral responses to circadian rhythm and sleep disruption between individuals. It may not always be possible to extrapolate findings from animal studies to humans (309), indicating a need for continued human research, especially in populations experiencing frequent circadian rhythm and sleep disruption. Furthermore, little research has explored such disruption in diseases like T2DM. There has also been little research on large populations likely experiencing circadian rhythm and sleep disruption. People living in China, for example, may be of particular interest because the country spans five geographical time zones, yet the entire nation follows Beijing time. It is plausible that chronic circadian rhythm and sleep disruption may incur some adaptations in the affected, although little research has addressed this to date.

Finally, behavioral and pharmaceutical interventions show promise in offsetting the adverse effects of circadian rhythm and sleep disruption. Some of the beneficial effects of these interventions may be independent of the circadian system and sleep, however. Because our understanding of the range of healthy phase relationships between the SCN and peripheral clock systems is poorly characterized, clarifying these relationships could help personalize prescription of chronobiotics, some of which still require human safety and efficacy studies. Thereafter, comparisons of these interventions are needed to evaluate which are most effective and in what circumstances.

Ultimately, we hope that mankind's historic fascination with the temporal world will continue to draw interest to the importance of the human timing system in all facets of health.
